# Olfactory preferences and chemical differences of fruit odors for *Aedes aegypti* mosquitoes

**DOI:** 10.1242/jeb.250305

**Published:** 2025-09-26

**Authors:** Melissa Leon-Noreña, Genevieve M. Tauxe, Jeffrey A. Riffell

**Affiliations:** Department of Biology, University of Washington, Seattle, WA 98195, USA

**Keywords:** Mosquito, Fruit attractants, Nectar, Fruit preference, Olfaction

## Abstract

Feeding on the nutrients from fruits and flowers is vital for mosquitoes and increases their lifespan, reproduction and flight activity. Olfaction is a key sensory modality in mediating mosquito responses to nutrient sources. Previous studies have demonstrated that fruits and flowers can vary in attractiveness to mosquitoes, with some sources preferred over others. However, how the attractiveness of different fruits relates to the chemical composition of their odor and the responses they evoke from the mosquito's peripheral olfactory system is still not understood. In this study, we used closely related fruit species and their cultivars to examine how changes in odor chemistry can influence the fruit's attractiveness to *Aedes aegypti* mosquitoes. Our results show that mosquitoes are attracted to the odors of certain fruits (*Mangifera indica*, *Prunus persica* and *Musa acuminata*), whereas others (*Pyrus communis* and *Citrus limon*) were not attractive. Chemical analyses of the odors showed that attractive fruits have distinct chemical profiles, and amongst closely related fruits, minor changes in the relative proportions of odor compounds can modify their attractiveness. By contrast, electroantennogram responses showed similar responses across different fruits. Selectively altering the chemical proportion of a single compound in an odor was sufficient to either increase or decrease its attractiveness to levels similar to those of its closely related congener. Our results demonstrate that mosquitoes are sensitive to the proportions of compounds in attractive odors, which have implications for the olfactory processing of complex odor sources, such as those from plants or blood hosts.

## Teaser

**Summary:** *Aedes aegypti* mosquitoes show specific and selective fruit odor preferences related to differences in the proportion of compounds in the odor.

## INTRODUCTION

Plant-nutrient feeding is a critical component for adult mosquitoes, with both males and females utilizing sources of nutrients from floral, fruit and extrafloral sources throughout their lives (Bradshaw et al., 2018; Brantjes and Leemans, 1976; Foster, 1995; Gu et al., 2011; Jhumur et al., 2007; Lahondère et al., 2020; Okech et al., 2003; Peach and Gries, 2016; Yuval, 1992). Plant nutrients are an essential part of the mosquito diet and the only source of food for males. Although females can use nutrient sources from blood meals, sugar and other nutrients from plants is still critical for other metabolic and behavioral processes, such as flight and oviposition (Foster, 1995; Yuval, 1992). Previous studies have shown that mosquitoes exhibit behavioral preferences to certain flowers and fruits, with some preferred over others (Manda et al., 2007; Müller et al., 2011; Nikbakhtzadeh et al., 2014; Yu et al., 2017). For example, in western Kenya, *Anopheles gambiae* mosquitoes exhibited distinct preferences for flowering plants, ranging from strong attraction to repellency or neutral behaviors (Manda et al., 2007; Müller et al., 2010). Similarly, in field experiments, *Aedes albopictus* mosquitoes were selectively attracted to certain, diverse flower species and rotting fruits (Müller et al., 2011), and in laboratory experiments, *Aedes aegypti* mosquitoes have shown clear preferences for specific flowering plants that also serve to increase their longevity (Chen and Kearney, 2015). However, the mechanisms by which mosquitoes discriminate between sources of plant nutrients are not clear.

Olfaction is a key sensory modality mediating the adult mosquitoes' ability to locate sources of food, including blood (De Obaldia et al., 2022; Zwiebel and Takken, 2004) and plant nutrients (Nikbakhtzadeh et al., 2014; Vargo and Foster, 1982; Von Oppen et al., 2015). Although ongoing work is shedding light on the relationship between human odor differences and relative attractiveness in mosquitoes (De Obaldia et al., 2022; Giraldo et al., 2023), few studies have examined comparative differences in the odor chemistry of plant nutrient sources and identified electrophysiologically active compounds in the odor (Barredo and DeGennaro, 2020; Jhumur et al., 2007; Lahondère et al., 2020; Nikbakhtzadeh et al., 2014; Nyasembe et al., 2018; Upshur et al., 2023). Flowering plants and fruits that are attractive to *An. gambiae* mosquitoes have been shown to emit electrophysiologically active compounds, including monoterpenes, sesquiterpenes and aliphatic compounds (Meza et al., 2020; Nyasembe et al., 2018). *Aedes aegypti* are selective in their antennal responses to monoterpenes, sesquiterpenes, esters, and aromatics (Lahondère et al., 2020; Nyasembe et al., 2018). However, the fruits and flowers that were less attractive, or neutral in their attractiveness, emitted many of the same compounds. It remains unclear which features of the odor – such as composition or intensity – may be driving these behavioral differences.

As odors are transported from the sources, their concentrations vary in space and time due to turbulent mixing by the wind (Riffell et al., 2008). Insects, including mosquitoes, can recognize behaviorally relevant odor sources despite these fluctuations in intensity (Dekker and Cardé, 2011; Murlis et al., 1992). For many insects, the proportion of certain key compounds in the odor is critical for recognizing attractive odor sources as the plume fluctuates in concentration, providing a chemical fingerprint for searching insects (Lahondère et al., 2020; Martin and Hildebrand, 2010). Examples of this phenomenon come from diverse insect species, including the sex pheromone system in Lepidoptera, where female moths emit a sex pheromone mixture of several key compounds at specific concentrations, the proportions of which are critical for the recognition by searching males (Martin et al., 2013; Roelofs and Cardé, 1977). Similar results have been shown in mosquitoes, where a floral species (*Platanthera obtusata*) attractive to *Aedes* spp. mosquitoes emitted odors dominant in aliphatic aldehyde compounds (e.g. nonanal and octanal) and low levels of monoterpenes (e.g. lilac aldehyde), whereas a sister species (*P. stricta*), pollinated by bees or moths, emits a fragrance similar to *P. obtusata* but dominated by lilac aldehyde, which is repellent to mosquitoes (Lahondère et al., 2020).

Differences in the proportions of various compounds in complex odors could explain the variation in mosquito attraction to sources of plant nutrients. The proportion of compounds in the odor of fruits differs widely, including those from closely related genera and even cultivars of the same species (Bouzayen et al., 2009; Du and Ramirez, 2022; Li et al., 2021). For example, the date palm – a favored fruit in mosquito lures – has different fruit cultivars that overlap in their odor composition, with some having a higher concentration of repellent terpenoid compounds, such as citronellol, whereas others have a higher concentration of attractive compounds, such as aliphatic aldehydes (Guido et al., 2011; Khalil et al., 2017). However, a systematic examination of the odors between closely related species and their relative attractiveness to mosquitoes has yet to uncover the relative importance of different odor features (composition, concentration or proportions) in attracting mosquitoes.

In this study, we took advantage of closely related fruits and their cultivars to examine the relationship between the odor composition, antennal olfactory responses and odor attractiveness to *Ae. aegypti* mosquitoes. Whole ripe and overripe fruits are an attractive and important nutrient source for mosquitoes, and mosquitoes have been shown to pierce the fruit exocarp to access the sugar and plant nutrients (Müller et al., 2011). Testing different fruit species – including those used in mosquito lures – and those of different cultivars allowed us to examine how odors overlapping in composition can evoke different levels of behavioral attraction. We present findings from (1) behavioral tests of different fruits and fruit varieties or cultivars, (2) analyses of fruit odor volatile compounds and emissions and their sources, (3) electrophysiological responses of the mosquito antennae to the fruit odors and (4) behavioral experiments showing how changes to a compound proportion in the fruit odor alter mosquito attraction. Using this integrative approach, we demonstrate that, for *Ae. aegypti*, attraction to fruit odors depends upon the chemical composition and the proportions of the odor, which have important implications for the olfactory processing of complex odors in *Ae. aegypti* mosquitoes, and future development of attractive lures.

## MATERIALS AND METHODS

### Mosquito rearing

*Aedes aegypti* (Linnaeus in Hasselquist 1762) mosquitoes used for behavior experiments were provided by BEI Resources (Manassas, VA, USA) and reared at the University of Washington (Seattle, WA, USA). In preliminary experiments, different *Ae. aegypti* lines (Rockefeller, Liverpool, Costa Rica and Puerto Rico, all from BEI Resources) were tested in their response to odor from mangoes (*Mangifera indica* ‘Tommy Atkins’), and all showed qualitatively similar levels of attraction, with approximately 55% to 85% of the mosquitoes attracted to the odor. Although the tested mosquitoes have remained in various insectaries for many generations, the responses to mango and other attractive fruits may suggest the fruit odors evoke an innate behavioral response. For the remainder of the experiments, we used the Rockefeller line, which showed consistent and robust responses to the fruit odor. Mosquitoes were maintained in an ACL2 insectary, per University of Washington Biological Use Authorization (BUA 0530-003), at 27°C, 70–80% relative humidity and a photoperiod cycle of 12 h:12 h light:dark. Eggs were hatched in plastic trays and deoxygenated with deionized water. Groups of 200 larvae were placed in covered trays containing tap water and fed with fish food (Hikari 129 Tropic 382 First Bites, Petco, San Diego, CA, USA). Pupae were grouped based on similar age and isolated in 16 oz containers (Mosquito Breeder Jar, BioQuip^®^ Products, Rancho Dominguez, CA, USA) and allowed to emerge. Experiments were conducted using adult mated mosquitoes 6–7 days old and fed 10% sucrose until 24 h before behavior experiments. Female mosquitoes were not blood-fed.

### Fruit selection

Fruits were selected owing to prior work on attracting mosquitoes and their use in mosquito toxic sugar baits, their presence in tropical and subtropical regions with endemic mosquito populations, and their availability to the study. We used intact fruits rather than fruit juices, concentrates or syrups that do not reflect the natural odor emissions (Joseph, 1970). Several of these fruits are similar in species or variety to those used in traps, and their corresponding studies and sources can be found in Table S1. The following fruit species and associated varieties or cultivars were tested: (1) mangoes: *Mangifera indica* ‘Ataulfo’, ‘Tommy Atkins’ and ‘Keitt,’; (2) guavas: *Psidium guajava* ‘Pink’ and ‘White’; (3) plums: *Prunus salicinia* ‘Santa Rosa’ and ‘Burgundy’; (4) peaches: *Prunus persica* ‘White Lady’ and ‘Monroe’; (5) nectarines: *Prunus persica* var. *nucipersica* ‘Fantasia’ and ‘Snow Queen’; (6) bananas and plantains: *Musa acuminata* ‘Cavendish’ and *Musa* × *paradisiaca*; (7) pears: *Pyrus communis* ‘Williams’ and *Pyrus pyrifolia* ‘Korean’; (8) date palms: *Phoenix dactylifera* ‘Barhi’ and ‘Medjool’; (9) tomatoes: *Lycopersicon esculentum*; and (10) lemons: *Citrus limon*. Fruits selected for each experiment were ripe and were inspected to have no signs of mold, bruises or damaged skin.

### Two-choice behavior assay

**Fig. 1.**
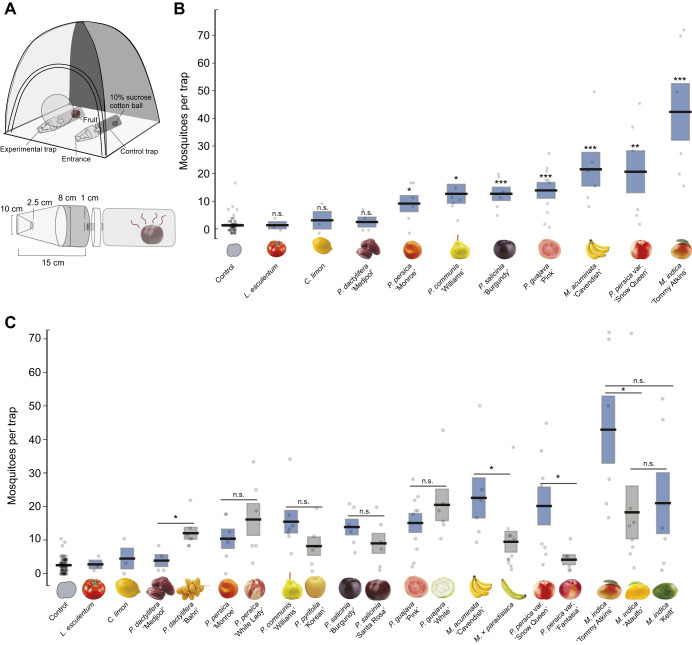
**Behavioral preferences of *Aedes aegypti* to fruit odors.** (A) Schematic of a two-choice behavior assay with control (10% sucrose cotton ball) and experimental trap. (B) Differences in behavioral attraction to different fruit species (see Materials and Methods for species names in full). The total number of mosquitoes inside the experimental fruit trap when provided with a choice between the fruit and the (no odor) control trap. The control (*n*=19) represents the number of mosquitoes counted inside the control trap across all fruits. Bioassays were also conducted, allowing the comparison of control traps in the fruit trials relative to cages containing two (no odor) control traps (*n*=9). No significant difference was measured between the two traps (Mann–Whitney *U*-test: *P*<0.05). (C) Closely related species, varieties and cultivars show significant variation in their attractiveness. Bars in A and B are means±s.e.m. Asterisks denote a significant difference between cultivars or closely related species (Mann–Whitney *U*-test: *P<0.05).

Three different experimental series were performed. The first series examined the mosquito preferences for different fruits and their paired fruit cultivar. To measure the preference of female and male mosquitoes towards fruit odors, a two-choice behavior assay was created consisting of a cage (Bugdorm, 60×60×60 cm) with two smaller traps (35 cm long) placed inside (Fig. 1A). Both traps contained an opaque glass chamber where the whole fruit or a negative control (10% sugar cotton ball) was placed. This chamber was connected by a polytetrafluoroethylene tube to a funnel trap, allowing odor from the fruit or control to passively enter the trap from the first chamber. The trap was constructed by placing a 10 cm diameter cone, with a 2.5 cm opening, into a plastic cup that was 8 cm in diameter and 15 cm in length. The chamber containing the fruit or controls was 13 cm in diameter and 25.5 cm in length. To keep from tipping, traps were placed horizontally on their sides such that the trap entrance was 5 cm above the ground; preliminary experiments with upright versus horizontally placed traps showed no difference in attractiveness to the flying mosquitoes. The experimental and control traps were set on opposite sides of the cage, and the placement of traps was randomized between replicates. Mosquitoes were starved of sugar, with water provided via soaked cotton balls 24 h before testing. Once released into the cages, mosquitoes were allowed to choose between experimental and control traps, and if attracted, readily navigated and flew into the opening of the trap. The experiment lasted for 48 h. The majority of mosquitoes entering the trap occurred within the 24–48 h period; the 48 h time allowed mosquitoes to visit the traps while keeping the fruit volatile organic compound (VOC) emissions constant and preventing changes owing to over-ripening. For each experimental replicate, approximately 75 mosquitoes were placed in each cage. The relative humidity from each trap was measured using a Sensirion 403-SEK-SENSORBRIDGE (Mouser Electronics, USA) to ensure that the differences in relative humidity between the experimental and control traps did not correlate with an increase in mosquito attraction (*r*=0.14, *P*=0.62). Between replicate trials, trap parts were disassembled and cleaned with 70% alcohol. Fruits were washed with an odorless soap (Tergazyme, Alconox Inc., USA) and allowed to air dry before each experiment. After 48 h, the total number of mosquitoes in each trap was counted and used in the statistical analyses. Mosquitoes that did not choose between traps were not included. For lemons and tomatoes, three replicate trials were performed, whereas experiments with all other fruits were replicated 6–9 times (Fig. 1B). Negative control trials, with no odor in either trap (only cotton balls with water), were run in parallel with each replicate.

The second series examined the behavioral preferences of mosquitoes for the fruit mesocarp versus exocarp. To examine the differences in mosquito attraction for each fruit part, an equal weight of exocarp or mesocarp was placed in their corresponding traps and tested against each other. The fruit exocarp was manually removed from the mesocarp using a peeler (Oxo Good Grips Y Vegetable Peeler, Oxo Corp., New York, NY, USA), weighed and then sectioned to the same mass as the exocarp. The two traps were then placed in the same cage, allowing a direct comparison of their relative attractiveness following protocols described above in the first series of experiments.

The third experimental series examined how changing the proportion of a single compound in the fruit odor influenced the attractiveness of the fruit. Three fruit species were used, and for each species, a single compound was added to the container to increase the proportion of that compound in the fruit's odor. The species and associated compounds were *M.* × *paradisiaca* spiked with isoamyl acetate, *P. persica* var. *nucipersica* (‘Snow Queen’) spiked with α-pinene and *M. indica* (‘Ataulfo’) spiked with 3-carene. These compounds are electroantennogram-responsive and important in plant nutrient detection (Lahondère et al., 2020; Meza et al., 2020; Leon-Noreña et al., 2025). Compounds were released using an infused silicon septum (Huber and Schiestl, 2022), allowing controlled emissions that simulate those of related fruits. Each compound was diluted in hexane (99%, Sigma-Aldrich), and the septa were placed in the solution for approximately 8 h in 10 ml glass scintillation vials at 20°C before being removed and air-dried in the fume hood before testing. The odorants were tested at two different concentrations (isoamyl acetate at 850 and 8500 µg ml^−1^, α-pinene at 8.5 and 85 µg ml^−1^, and 3-carene at 85 and 850 µg ml^−1^); a septum soaked in hexane was used as the control. The lower concentration allowed us to simulate the emission rates of the compounds in the different congeners, whereas the higher concentration allowed us to determine whether concentration-dependent effects (linear or non-linear) occurred.

In these experiments, we simultaneously tested male and female mosquitoes. We found no significant differences between the two sexes in their relative attraction to the fruit odors (Mann–Whitney *U*-test: *P*=0.22), although slightly higher numbers of female mosquitoes were attracted to the fruit odor traps overall (ratio of 1:1.2 male-to-female). Additional control experiments compared male- and female-only trials with those with both sexes and showed no significant difference between experiment types in the number of mosquitoes attracted to the fruit traps (Mann–Whitney *U*-test comparing male-only versus both sexes: *P*=0.54; Mann–Whitney *U*-test comparing female-only versus both sexes: *P*=0.78).

### Fruit headspace collection

Headspace collections were performed to identify VOCs emitted from each fruit. Each intact fruit was washed with odorless soap (Tergazyme) and air-dried before odor collection. The intact fruit was then weighed and placed inside a nylon bag (Reynolds, USA) for 24 h for volatile collection. Two PTFE tubes (¼″ ID×5/16″ OD, Fluorostore) were inserted into the bag: one provided air through a charcoal-filtered Pasteur pipette into the bag (1 l min^−1^), and the other vacuumed air from the bag (1 l min^−1^) into a borosilicate Pasteur pipette containing 100 mg of Porapak powder Q 80-100 (Waters Corporation, USA) and deactivated glass wool (Thermo Fisher Scientific, USA).

VOCs were eluted from each sorbent cartridge in 600 µl of hexane (99%, Sigma-Aldrich) and stored in 2 ml amber vials at −80°C until analysis. Although Porapak Q does not efficiently capture small molecules such as CO_2_, or semi-volatile fatty acids, it does capture diverse volatile compounds greater than 100 Da including monoterpenes, aromatic, aliphatic aldehydes, alkanes, esters and short-chain alcohols. A series of preliminary experiments were conducted to maximize the capture of diverse compounds, where fruit headspace collections were conducted for different lengths of time (4, 8, 12 and 24 h) and using different amounts of Porapak Q (30, 50, 100 and 200 mg). The 24-h period, using 100 mg of Porapak Q, enabled us to capture the greatest diversity of VOCs across different fruit species.

For the exocarp and mesocarp odor collection, an equal mass of each fruit part was placed inside a nylon bag and sampled for 24 h. Briefly, the fruit exocarp was manually removed from the mesocarp using a peeler (Oxo Good Grips Y Vegetable Peeler) and weighed. The mesocarp was then sectioned to be the same mass as the exocarp before headspace sampling.

For every series of sample collections, a negative control (empty bag) was run in parallel. In addition, contaminants from the solvent, the sample matrix and the GC column were also identified and removed from the datasets.

### Fruit odor chemical identification and quantification

Analyses were conducted using an Agilent 7890A GC and 5975C Network Mass Selective Detector (Agilent Technologies, Santa Clara, CA, USA). A DB-5MS GC column (J&W Scientific, Folsom, CA, USA; 30 m, 0.25 mm, 0.25 μm) was used with helium as a carrier gas at a constant 1 cc min^−1^ flow. Automated injections of 3 µl for each sample were inserted into the GC using a GS 7693 autosampler (Agilent Technologies) in spitless mode (220°C) with the oven temperature held at 45°C for 6 min, followed by a heating gradient of 45°C to 220°C at 10°C min^−1^, and then held isothermally for 6 min. Chromatogram peaks were manually integrated using ChemStation software (Agilent Technologies), filtered and tentatively identified by the online NIST library with confirmation matches >70%. Putative identifications were verified by calculated Kovats retention indices and comparison to synthetic standards. The concentrations of compounds of interest were determined by comparison to standard curves of synthetic standards measured from 0.5 ng µl^−1^ to 1 µg µl^−1^. Total odor emission rates (ng h^−1^) were determined from the quantified odor compounds and normalized to the mass of each fruit. Relationships among the fruit samples' odor composition were plotted and analyzed using a non-metric multidimensional scaling (NMDS) analysis.

### Electroantennogram experiments

Electroantennograms (EAGs) were performed using similar procedures to Lahondère et al. (2020). Antennae were prepared by dissecting the *Ae. aegypti* heads from the insect and the distal tip was removed with tenotomy scissors. The head was placed on the reference electrode with the antennae tips placed on the tip of the Syntech EAG recording probe using Spectra 360 electrode gel (Parker Labs, Fairfield, NJ, USA), with the base of the antennae placed on the reference electrode so that the electrodes measured the bulk sum of electrical activity across the antennae. The EAG electrodes with mounted antennae were placed before a continuous air stream (1000 ml min^−1^ flow; Gilmont flowmeter, Gilmont Industries/Barnant Company, Barrington, IL, USA) at 25°C room temperature. The electrodes were connected to a Syntech headstage, connected to an IDAC-4 (Ockenfels Syntech GmbH), allowing 60 Hz noise reduction and filtering. Antennal deflections were counted as responses for a fruit odor if they were 1.5 standard deviations above the noise floor of the antennal activity and occurred within a 0.5-s window of the odor release. The threshold was individually calibrated based on differing levels of signal and noise in each preparation. For each odor stimulus, eight to twenty-six 5- to 7-day-old female *Ae. aegypti* mosquitoes were tested. As in previous studies (Riffell et al., 2013; Lahondère et al., 2020), Pasteur pipettes containing the headspace collections were prepared by aliquoting 50 µl onto a piece of filter paper (3 mm by 40 mm) (Whatman Inc., Clifton, NJ, USA). The hexane solvent was allowed to dry for 7 min before the filter paper was inserted in a Pasteur pipette to deliver the fruit odor. Each fruit odor and control stimulus [hexane solvent control, and positive control stimulus 3-methyl-1-butanol (hereafter, isopentanol), diluted at 1% v/v in hexane] was presented randomly. EAG response amplitudes were quantified offline using the Autospike software and normalized to the positive control stimulus.

### Statistical analysis

Statistical analyses were conducted using MATLAB software, v2020b (MathWorks, Natick, MA, USA). The response variable for the behavioral preference assays was the number of mosquitoes in each trap. Non-parametric Mann–Whitney *U*-tests were deemed suitable, given the lack of normality for within-genera and within-species comparisons. A significant criterion of 0.05 was used for all statistical testing, except those involving multiple comparisons where the criterion was adjusted. A Kruskal–Wallis test was used to statistically test the relationship between the compounds identified in the fruit odors and the attractiveness of the fruits and to compare the odor emissions.

NMDS analyses were performed to analyze variations in odor composition among fruit varieties and species. For these multivariate analyses, we first coded all identified compounds as either present (1) or absent (0) to examine the dissimilarity between fruits, and then constructed a matrix of Bray–Curtis dissimilarities calculated on the relative proportions of the odor compounds. An analysis of similarity (ANOSIM) was performed on the proportion data used in NMDS. ANOSIM is a non-parametric permutation analysis used to assess the similarity between multiple groups regarding the compounds within the odor. To evaluate the clustering in the NMDS, an iterative *k*-means clustering was performed on the proportional dataset. The number of clustering centroids was determined using the elbow method via computing the distortions under different cluster numbers, where the best cluster number corresponded to 90% of the variance explained (defined as the ratio of the between-group variance to total variance).

## RESULTS

### Behavioral response to fruit odors

As the first step in examining differences across fruit odors, we examined mosquito responses to the negative (no odor) control, run in parallel for each treatment and replicate trial (Fig. 1). Across all two-choice behavioral trials, there was no significant difference in the number of mosquitoes attracted to the control trap (Kruskal–Wallis test: χ_15,99_=19.3, *P*=0.19), or cages containing two control traps (Kruskal–Wallis test: χ_1,119_=1.5, *P*=0.22). On average, 2.4±0.25 (mean±s.e.m.) mosquitoes per trial were attracted to the control trap. By contrast, across all the fruits tested, there were significantly greater numbers of mosquitoes in the baited traps containing the fruits than in the control traps (Kruskal–Wallis test: χ_1,199_=73.88, *P*<0.0001), with 14.0±1.2 mosquitoes per trap.

There was significant variation in the attractiveness between fruit odors. *Mangifera indica* (‘Tommy Atkins’) was the most attractive, with a mean of 41.1±9.1 mosquitoes per trap. *Prunus persica* var. *nucipersica* (‘Snow Queen’), *Psidium guajava* (‘White’), *Musa acuminata*, *Prunus salicinia* (‘Santa Rosa’), *Prunus persica* (‘Monroe’) and *Pyrus communis* (‘Williams’) elicited similar levels of attraction, with approximately 15±1.8 mosquitoes per trap. By contrast, *P. dactylifera* (‘Medjool’), *L. esculentum* and *C. limon* were the least attractive (3.7±1.7, 2.6±1.2 and 4.3±2.9 mosquitoes per trap, respectively), and not significantly different from the negative controls (Kruskal–Wallis test with multiple comparisons: *P*>0.98; Fig. 1B).

To examine how odors from closely related fruits may differ in their attractiveness, we tested the cultivars, varieties and closely related species of fruits (Fig. 1C). We observed significant differences when we examined attraction at the level of varieties and cultivars (Kruskal–Wallis test: χ_1,181_=108.6, *P*<0.0001). For example, the odor of *M. indica* (‘Tommy Atkins’) was significantly more attractive than that of the *M. indica ‘*Ataulfo’ cultivar (Mann–Whitney *U*-test: *P=*0.004). There was a similar effect in the nectarines (*P. persica* var. *nucipersica*), with the ‘Snow Queen’ cultivar attracting 4-fold more mosquitoes than the ‘Fantasia’ cultivar (Mann–Whitney *U*-test: *P*=0.04) (Fig. 1C). There was also a significant difference in the attractiveness of the banana (*M. acuminata*) and plantain (*M.* × *paradisiaca*) odors (Mann–Whitney *U*-test: *P*=0.02), with *M. acuminata* attracting almost twice as many mosquitoes as *M. *× *paradisiaca* (21.9 and 9.1 mosquitoes per trap for *M. acuminata* and *M. *× *paradisiaca*, respectively). There was no difference in the numbers of attracted mosquitoes for the *P. guajava* cultivars (‘Pink’ versus ‘White’), other pairs of cultivars in the *Prunus* group, or between *Pyrus* species (Mann–Whitney *U*-tests: *P*>0.05). Although *P. dactylifera* varieties attracted relatively few mosquitoes (11.5 and 3.5 for ‘Bahri’ and ‘Medjool’, respectively), they were significantly different from one another (Mann–Whitney *U*-test: *P*=0.01).

### Chemical analysis of fruit odors

**Fig. 2.**
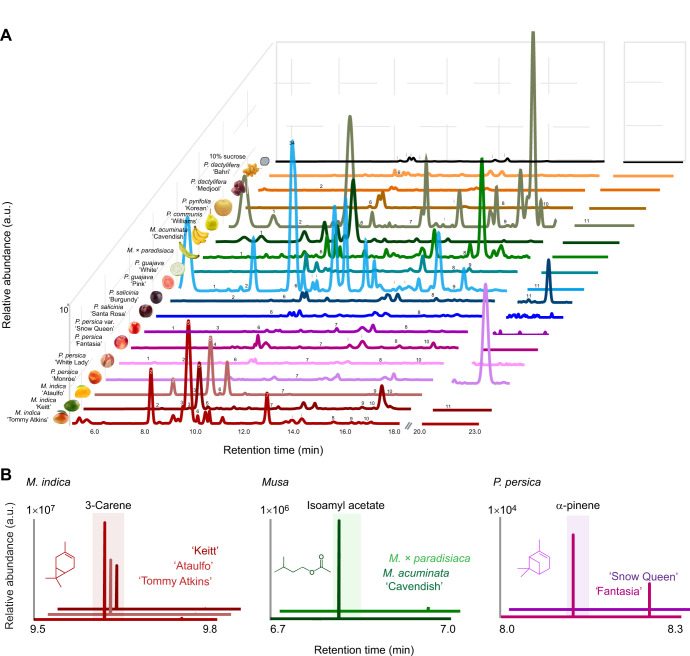
**Ion chromatograms and chemical profiles of fruit odors.** (A) Representative gas chromatography-mass spectrometry (GC-MS) ion chromatograms for each corresponding fruit and associated compounds of interest. Numbers indicate: (1) isoamyl acetate, (2) α-pinene, (3) ethyl hexanoate, (4) hexen-1-ol-acetate, (5) 3-carene, (6) limonene, (7) ethyl octanoate, (8) tetradecane, (9) caryophyllene, (10) α-farnesene and (11) methyl hexadecanoate. Contaminants are denoted by *i.* (B) Differences in the concentrations of select bioactive volatiles between species and cultivars for *Mangifera indica* and 3-carene (left), *Musa* and isoamyl acetate (middle) and *Prunus persica* var. *nucipersica* (right).

**Fig. 3.**
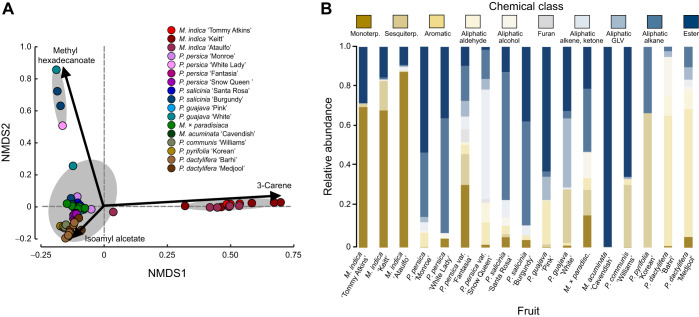
**Chemical type proportions and multivariate analysis of fruit odors.** (A) Non-metric multidimensional scaling (NMDS) biplot of the chemical composition of all fruit groups and varieties are represented by color and corresponding shade. Analysis of the odor compositions are significantly different between fruits (ANOSIM: *R*=0.8417, *P*=0.001), with significantly different clusters denoted by gray ellipses. Labeled arrows denote the chemical compounds dominating the different axes. (B) Fruit odor profile demonstrating the ratio of different classes of compounds for each fruit group and cultivar, including: monoterpenes, sesquiterpenes, aromatics, aliphatic aldehydes, aliphatics alcohols, furans, aliphatic alkenes and ketones, aliphatic green leaf volatiles (GLVs), aliphatic alkanes and esters.

VOCs were identified from, and average emission of fruit odor was determined for each of the 19 fruits used in behavioral tests. Across all samples, we identified 150 compounds, including 30 terpenoids, 20 aromatics, two sulfurs, two furans and 96 aliphatic compounds (Table S2). There were significant differences in the emission rates and total number of odor compounds among the fruit samples (Kruskal–Wallis test: χ_16,74_>53.84, *P*<0.001), but there was no significant correlation between these factors and the fruits' attractiveness (Spearman correlation: ρ<0.22, *P*>0.38). There were also qualitative differences among the sampled fruits. For instance, *M. indica* cultivars emitted a diverse suite of terpenoid compounds (Fig. 2; Table S2), whereas *P. guajava* cultivars were enriched in short-chain aliphatic compounds, such as hexenol acetate and ethyl butyrate. Members of the *Prunus* group (plums, peaches and nectarines) slightly differed in their odor composition, with plums and peaches emitting higher amounts of aliphatic alkanes (e.g. hexadecane and heptadecane), whereas the nectarines emitted more sesquiterpenes and aromatic compounds (e.g. α-farnesene and benzaldehyde, respectively). *Musa acuminata* odor was composed of aliphatic esters and short-chain compounds (e.g. isoamyl acetate and acetic acid), whereas *M.* × *paradisiaca* odor was enriched in alkanes and monoterpene compounds (e.g. hexadecane and limonene). Both pear species emitted odors enriched in the sesquiterpene α-farnesene, but *P. communis* emitted more aliphatic esters, whereas *P. pyrifolia* emitted more sesquiterpenes (Fig. 3; Table S2).

To analyze the variability generated by the 150 compounds across the 19 fruit species, varieties and cultivars, we conducted a multivariate analysis (NMDS) using the proportion of compounds in the fruit odors (Fig. 3). This analysis also found a significant difference between fruit odors (ANOSIM: *R*=0.6963, *P*=0.001). The attractive white nectarines (*Prunus persica* var. *nucipersica* ‘Snow Queen’) were close to the other *Prunus* species and varieties and to the other fruit species (Fig. 3A). By contrast, *M. indica* cultivars occupied a distinct area along NMDS1 and NMDS2. By plotting individual volatiles in the same NMDS space, we found that the monoterpene 3-carene was distributed along the NMDS1 axis, whereas the sesquiterpene caryophyllene, the ester isoamyl acetate and the aliphatic compound hexadecane were distributed along the NMDS2 axis (Fig. 3A). Between the closely related species and fruit cultivars, their proportions of compound types in the odors also showed significant variation, especially their proportion of monoterpenes. For instance, *P. guajava* cultivars differed in their terpenoid and aromatic compound proportions, *P. persica* var. *nucipersica* cultivars differed in their proportion of monoterpene and aromatic compounds, and *M. indica* cultivars differed in their relative amounts of terpenes, including 3-carene (Fig. 3B).

### Antennal olfactory responses to the fruit odors

**Fig. 4.**
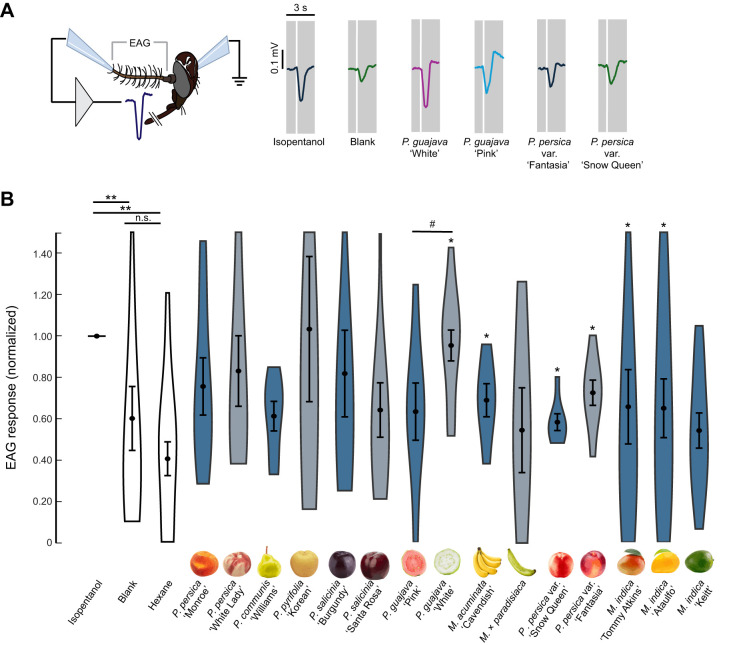
**Electroantennogram responses to the fruit odors.** (A) Experimental setup for the electroantennogram (EAG) experiments (drawing courtesy of M. Stensmyr). Traces are the individual EAG responses to an olfactory stimulation (0.5 s) showing the responses to isopentanol (+control), clean air blank (–control), guava varieties (*Psidium guajava* ‘White’ and ‘Pink’) and nectarine varieties (*Prunus persica* var. *nucipersica* ‘Fantasia’ and ‘Snow Queen’). (B) Violin plots of EAG responses across the tested olfactory stimuli. Statistical analyses were performed with the normalized data (relative to isopentanol control in each preparation). Plots are means±s.e.m. for each stimulus (*n*=8–25 mosquitoes per odor stimulus). Asterisks denote significant responses compared with the solvent control (Mann–Whitney *U*-test: *P<0.05). Comparing responses between varieties and closely related fruit species showed that only *P. guajava* varieties elicited significantly different responses (Mann–Whitney *U*-test: ^#^*P*=0.001).

The select preference of *Ae. aegypti* mosquitoes for the odors of certain fruits and fruit varieties motivated us to examine whether their antennae respond differently to those odors. We performed EAG recordings to measure the summed response of olfactory sensory neurons on the mosquito antennae to a panel of fruit headspace collections (Fig. 4). Results from these experiments showed that fruit headspace collections evoked stronger EAG responses relative to the blank odor cartridge and solvent controls (Kruskal–Wallis test: χ_17,274_=95.66, *P*<0.0001; Fig. 4). *Mangifera indica*, *P. guajava*, *P. persica*, *Prunus persica* var. *nucipersica* and *Musa* spp. cultivars evoked significantly stronger responses than the solvent control (Dunn–Šidák test: *P*<0.02), but *P. salicinia* and *Pyrus* spp. were not different from the controls (Dunn–Šidák test: *P*>0.10). Among each pair of related species and cultivars, only the *P. guajava* cultivars evoked significantly different responses from each other, with the ‘White’ cultivar evoking stronger responses than the ‘Pink’ (Mann–Whitney *U*-test: *P*=0.02). There was no significant correlation between the EAG responses and fruit odor emission rates (Spearman correlation: ρ=−0.48, *P*=0.06), nor the EAG responses and the number of mosquitoes attracted to the odors (Spearman correlation: ρ=0.05, *P*=0.85).

### Source of dominant volatiles in the fruit odor

**Fig. 5.**
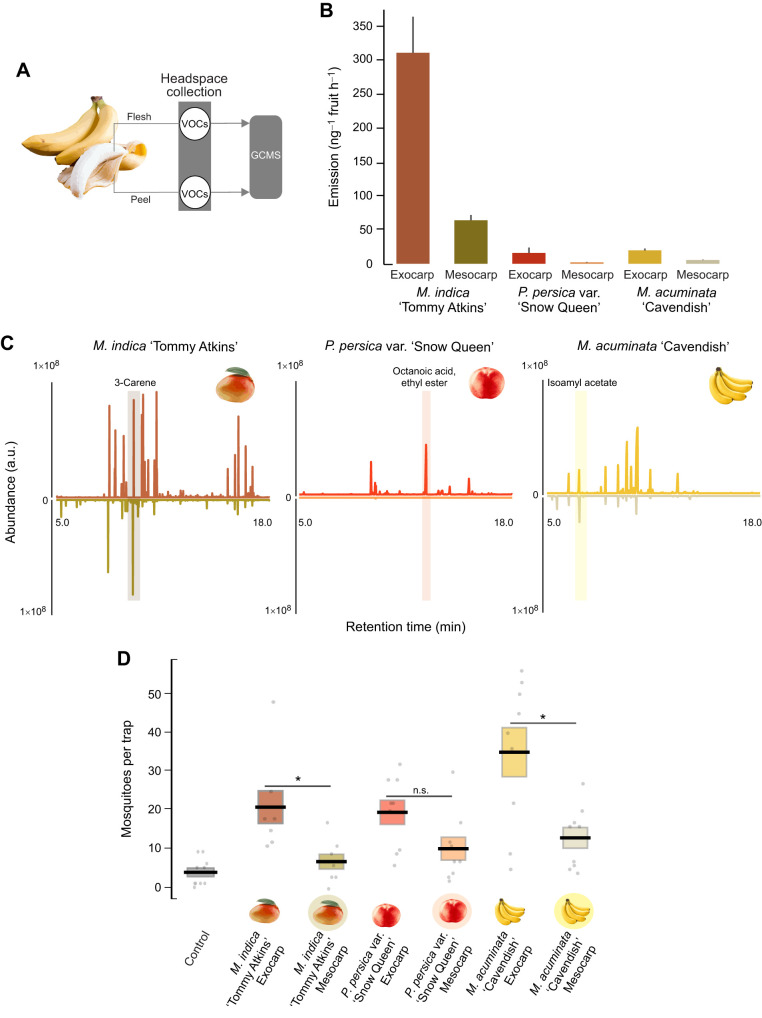
**Volatile source emissions from fruits.** (A) Schematic of the headspace collections from the exocarp (peel) and mesocarp (pulp). VOCs, volatile organic compounds. (B) Emission of exocarp and mesocarp of three fruits: *Mangifera indica* ‘Tommy Atkins’, *Prunus perspica* var. *nucipersica* ‘Snow Queen’ (white nectarine) and *Musa acuminata* (banana). Bars are means±s.e.m. (C) Ion chromatograms of the exocarp and mesocarp of the same three fruits. (D) Results from the behavioral assays directly testing the attractiveness of the mesocarp versus the exocarp for the three fruits. Bars are means±s.e.m. Asterisks denote a significant difference between the exocarp and mesocarp for the different fruits.

To evaluate the potential sources of the different VOCs emitted from the fruits, we sampled the headspace of the exocarp (peels) and mesocarp (pulp) of three of the most attractive fruit species: *M. indica* (‘Tommy Atkins’), *P. persica* var. *nucipersica* (‘Snow Queen’) and *M. acuminata* (Fig. 5A). For all three fruits, there was a significant difference in the odor between the fruit exocarp and mesocarp (Kruskal–Wallis test: χ_5,17_=19.44, *P*=0.001), with the exocarp emitting 3.7- to 8.9-fold higher levels of VOCs compared with the mesocarp (Fig. 5B). These quantitative differences in odor emission were also reflected in differences in chemical composition and proportions between the parts of the fruit. Examples of these differences can be found in *M. indica* (Figs 1B, 3B), where terpenes dominated the odor emissions of the exocarp and whole fruit, whereas ketones and short-chain alcohols were the dominant compounds in the odor of the mesocarp (e.g. cyclopentanone and 1-hexanol). These differences in the composition and compound proportions of the exocarp and mesocarp odors were also found in *P. persica* var. *nucipersica* and *M. acuminata*. The *P. persica* var. *nucipersica* mesocarp odor was dominated by cyclopentanone, whereas fatty acid esters such as ethyl hexanoate dominated the exocarp odor. *Musa acuminata* mesocarp odor included many different compounds, including 2-pentanol acetate and isopentyl isobutyrate, whereas its exocarp odor was dominated by isoamyl butanoate and isoamyl acetate (Table S2).

Behavioral tests of the fruit mesocarp and exocarp showed differences in their level of attractiveness to the mosquitoes (Fig. 5C). The exocarps of both *M. acuminata* and *M. indica* (‘Tommy Atkins’) attracted significantly higher numbers of mosquitoes than their mesocarps (Mann–Whitney *U*-test, *P*<0.02), whereas for *P. persica* var. *nucipersica* (‘Snow Queen’), the exocarp attracted higher numbers of mosquitoes, but it was not statistically different from the mesocarp (Mann–Whitney *U*-test, *P*=0.06).

### Behavioral effects of altering the compound proportions in the fruit odors

The differences in the attractiveness of closely related fruits motivated us to ask whether changing the proportion of a single compound was sufficient to change the fruit's attractiveness. We took advantage of three fruits – *M.* × *paradisiaca*, *P. persica* var. *nucipersica* (‘Snow Queen’) and *M. indica* (‘Ataulfo’) – which vary in attractiveness compared with their paired species or cultivar. For *P. persica* var. *nucipersica*, the difference between the attractive and less attractive cultivars was related to the proportion of the ⍺-pinene in the odor (0.02 and 0.003 ng h^−1^ fruit^−1^ for ‘Fantasia’ and ‘Snow Queen’ cultivars, respectively), whereas for *M.* × *paradisiaca* it was reflected in isoamyl acetate (1.08 and 8.73 ng h^−1^ fruit^−1^ for *M.* × *paradisiaca* and *M. acuminata*, respectively) (Fig. 1B). For *M. indica*, differences in emissions of 3-carene differed between less attractive ‘Ataulfo’ and attractive ‘Tommy Atkins’ cultivars (12.4 and 32.7 ng h^−1^ fruit^−1^, respectively) (Fig. 1B). We increased the emission of these compounds using an infused silicon septa, allowing us to test two different concentrations in the context of the fruit odor.

**Fig. 6.**
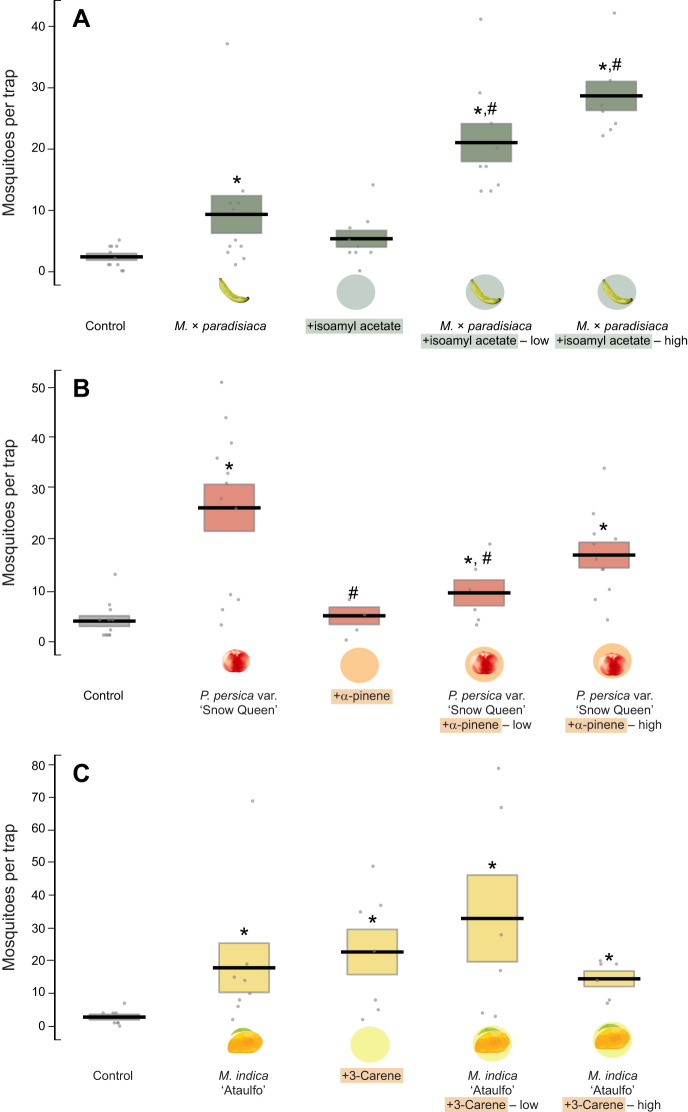
**The effects of altering different compound proportions in *Musa*** × ***paradisiaca, Prunus persica* var. *nucipersica* and *Mangifera indica* odors.** (A) The number of mosquitoes attracted to traps emitting *M.* × *paradisiaca* fruit odor, with the addition of either a solvent control or isoamyl acetate at two concentrations to simulate the proportion of isoamyl acetate emitted from the more attractive *M. acuminata* (low concentration, 8.7 ng h^−1^), or a 10-fold higher concentration (high concentration, 87.3 ng h^−1^). The isoamyl acetate was also tested alone as a control at 87.3 ng h^−1^. (B) Same as in A, but testing *P. persica* var. *nucipersica* (‘Snow Queen’) fruits with the increased addition of α-pinene to simulate the proportions of the less attractive ‘Fantasia’ cultivar (0.02 ng h^−1^) and a 10-fold higher concentration (0.2 ng h^−1^). The α-pinene was also tested alone as a control at 0.2 ng h^−1^. (C) Same as in A, but testing the less attractive *M. indica* ‘Atualfo’ fruits with the increased addition of 3-carene to simulate the proportions of the more attractive ‘Tommy Atkins’ cultivar (32.7 ng h^−1^) and a 10-fold higher concentration (327.4 ng h^−1^). The 3-carene was also tested alone as a control at 327.4 ng h^−1^. For each experimental replicate, a negative control trap was run simultaneously. Bars are means±s.e.m. Asterisks denote a significant difference between the fruit treatments and negative solvent control, and hatch symbols denote a significant difference between the odorant alone or odorant+fruit treatments and the fruit alone (Mann–Whitney *U*-test; *^,#^*P*<0.05).

*Musa* × *paradisiaca* spiked with two different concentrations of isoamyl acetate attracted significantly more mosquitoes than *M.* × *paradisiaca* alone (Mann–Whitney *U*-tests: *P*<0.01) (Fig. 6A), attracting similar numbers of mosquitoes to *M. acuminata* (Mann–Whitney *U*-tests: *P*=0.09). Moreover, compared with the negative control, isoamyl acetate alone at the higher concentration attracted approximately the same number of mosquitoes (Mann–Whitney *U*-tests: *P*=0.08).

By contrast, for the attractive *P. persica* var. *nucipersica* (‘Snow Queen’), the addition of α-pinene to the fruit odor caused fewer mosquitoes to be attracted to the traps, although only the lower concentration was significantly different (Mann–Whitney *U*-test, *P*=0.04) (Fig. 6B). Comparing the number of mosquitoes attracted to *P. persica* var. *nucipersica* (‘Snow Queen’) spiked with ⍺-pinene with the less attractive ‘Fantasia’ cultivar, which naturally emits higher levels of ⍺-pinene, showed no significant differences in their level of attraction (Mann–Whitney *U*-test: *P=*0.43). The experimental treatment of α-pinene alone was not statistically different from the negative control, having similar numbers of mosquitoes (Mann–Whitney *U*-test, *P*=0.50).

Finally, for the less attractive *M. indica* (‘Ataulfo’), 3-carene had a concentration-dependent effect. At a low concentration that simulated the emissions of the ‘Tommy Atkins’ cultivar, the odor attracted a higher number of mosquitoes, but it was not statistically different than the ‘Ataulfo’ fruit odor alone (Mann–Whitney *U*-test: *P*=0.50) (Fig. 6C). Conversely, fewer mosquitoes were attracted when 3-carene was added to the fruit odor at a higher concentration. When compared with the negative control, 3-carene alone at the high concentration was significantly attractive (Mann–Whitney *U*-test: *P*=0.005).

Across all treatments, there was no significant difference in the number of mosquitoes caught in the negative control traps (Mann–Whitney *U*-tests: *P>*0.10). Taken together, these results suggest that a change in the proportion of a single compound in the fruit odor, simulating the chemical composition of the tested fruit pair, can sometimes significantly alter the attractiveness of a fruit.

## DISCUSSION

Motivated by the dearth of studies examining how differences in the odors of plant nutrient sources influence mosquito attraction, we examined the preference of *Ae. aegypti* mosquitoes to the odors of closely related fruits and their cultivars. Our results show that both male and female *Ae. aegypti* mosquitoes have attractive preferences for specific fruit species and varieties, including *M. indica*, *P. perspica* var. *nucipersica*, *P. guajava* and *M. acuminata*, whereas other fruits (*P. communis* and *C. limon*) were not attractive. Although the chemical profile can differ between disparate species – for example, *Musa* spp. compared with *Mangifera* spp. – the differences within species predominantly reflect changes in the proportion of compounds in the odor. Similar to prior work that tested different flower odors (Lahondère et al., 2020), our results show that the proportion of compounds in fruit odors can have a strong behavioral effect.

Mosquitoes are attracted to diverse flowers, fruits and honeydew as plant nutrient sources (Athen et al., 2020; Lahondère et al., 2020; Peach et al., 2019; Yalla et al., 2023). However, various studies, including those in the laboratory, semi-field and field, have shown that plant nutrient sources can be differentially attractive to mosquitoes, with some plants highly attractive and others eliciting little to no attraction (Gary and Foster, 2004; Müller et al., 2011). In an elegant series of experiments, Nyasembe et al. (2018) examined the putative feeding preferences of field-caught mosquitoes in Kenya using DNA bar-coding and found that *Anopheles gambiae* s.s. mosquitoes may predominantly have fed on a subset of plants in the environment, such as *Senna alata* (Fabaceae), *Ricinus communis* (Euphorbiaceae) and *Parthenium hysterophorus* (Asteraceae), whereas *Ae. aegypti* mosquitoes may have fed on *Senna uniflora* (Fabaceae) and *Hibiscus heterophyllus* (Malvaceae) (Nyasembe et al., 2018). Similar results have shown that diverse mosquitoes preferentially feed from diverse flowering plants (Lahondère et al., 2020; Manda et al., 2007; Müller et al., 2010, 2011; Yu et al., 2017). Research examining the preferences of *Culex pipiens pallens* mosquitoes indicated that they are differentially attracted to several flowering plant species, including *Tagetes erecta* (Asteraceae) and *Catharanthus roseus* (Apocynaceae) (Yu et al., 2017). *Aedes albopictus* adults were differentially attracted to *Tamarix chinensis* (Tamaricaceae), *Ziziphus spina-christi* (Rhamnaceae), *Prosopis farcta* (Mimosaceae) and other plant families (Müller et al., 2011). The diversity in plant species and families used as nutrient sources makes it difficult to identify specific plant groups mosquitoes may feed on, and instead may reflect similarities in the odor profiles of the flowering plants, or local plant abundances that mosquitoes can adaptively utilize.

In contrast to the growing body of work using flowering plants, research on mosquito preference for intact fruit odors has received comparatively less attention. Examples include the mosquito *Culex pipiens pallens*, which showed attraction to the odors of peach and melon (*Amygdalus persica* and *Cucumis melo*, respectively) but was less attracted to pear (*Pyrus bretschneideri*) (Yu et al., 2017). In field trials, male and female *Ae. albopictus* mosquitoes showed attraction to sabra and figs (*Opuntia ficus indica* and *Ficus carica*, respectively) but not undamaged pomegranate (*Punica granatum*) (Müller et al., 2011). Research from the present study shows that attraction can also vary between closely related fruit species and cultivars. Although we only tested male and female mosquitoes of one species (*Ae. aegypti*), previous work in other mosquito species, such as *An. gambiae*, has also demonstrated attraction to different cultivars of the same species, including *M. indica* ‘Kent’ (Meza et al., 2020).

This strong preference by mosquitoes for specific fruit odors also raises questions about the relatedness and differences in the odor profiles between plant nutrient sources. Although the chemical composition of the sources of plant nutrients can differ, many of the attractive odors share the presence of compound types in their profile, including various isomers of pinene, myrcene, terpinolene, linalool and linalool oxide, and caryophyllene (Lahondère et al., 2020; Nikbakhtzadeh et al., 2014; Tenywa et al., 2017). Other compound types, including aliphatic aldehydes and esters, have also been shown to be important for mosquito detection of plant nutrient sources (McGovern et al., 1970). Similar compounds are found in the headspace of many fruits tested in this study, including mango, peach and nectarine (Figs 2 and 3). *Musa acuminata* is another fruit that is attractive to *Ae. aegypti* mosquitoes and emitted an odor that was dominated by aliphatic ester compounds, including 2-pentyl acetate, isoamyl acetate and isoamyl butyrate (Table S2), some of which were also emitted by the attractive mango and guava fruits. Nonetheless, across these similarities, the differences in odor compositions between closely related species and varieties may provide insight into the compounds that decrease the attractiveness of the fruits. For example, *P. persica* var. *nucipersica* ‘Snow Queen’ emits lower amounts of monoterpenes, including α-pinene, than the less attractive ‘Fantasia’ cultivar. Increasing the concentration of α-pinene in the odors decreased their attractiveness (Fig. 6). Beyond monoterpenes, *P. salicinia ‘*Burgundy’ emits higher levels of esters and aliphatic aldehydes, known attractants to mosquitoes (Bosch et al., 2000; Takken and Knols, 1999), compared with the ‘Santa Rosa’ cultivar, whereas for peaches, the *P. persica ‘*Monroe’ emits higher levels of benzenoid compounds than the ‘White Lady’ cultivar. These benzenoid compounds have been implicated as both attractants (acetophenone) (Afify and Potter, 2020) and repellents (benzaldehyde) (Zhang et al., 2022) in mosquitoes, and the concentration and proportion of these compounds in the odor may be critical for the valence of the mosquito's behavior. Besides the specific compounds in the odors, the differences in attraction between cultivars and varieties may be related to the proportion of compounds in the odors (Figs 2 and 3). An important aspect of this study is the use of Porapak Q adsorbent to collect fruit odors. This adsorbent, although ideal for diverse volatile types, will not collect small molecular weight compounds (<100 Da) nor efficiently collect high molecular weight polar compounds, such as fatty acids. Future work will need to use alternate adsorbent methods, such as solid-phase microextraction fibers with polydimethylsiloxane/divinylbenzene coating, to allow the capture and identification of polar and semi-volatile compounds in the odors, as well as to characterize the different chiral compounds in these odors. Despite these potential caveats, our work quantified a diverse panel of compounds in the fruit odors and showed that manipulating the concentration of a single compound in an attractive headspace was sufficient to lower the fruit's attractiveness to levels similar to its non-attractive cultivar.

The differences in behavioral attraction were not reflected in the antennal olfactory responses to the fruit odors. Results from our EAG experiments showed that the mosquito antennae evoked strong responses to many of the fruit odors compared with the negative control, but that only one pair of varieties, from *P. guajava*, elicited significantly different responses (Fig. 4). A weakness of the EAG method is that it reflects the bulk sum of all receptor potentials in the antenna, and although it may reflect the olfactory drive to the antennal lobe in the brain, it may not provide explicit determination of how features of the odor (e.g. composition, proportions and concentrations) are encoded to mediate behavior. Instead, we suggest that similar EAG responses with differing behavioral outcomes may reflect the downstream processing in the mosquito's brain. Prior work has shown that glomeruli in the mosquito antennal lobe are sensitive to subtle differences in the proportion of compounds in closely related floral odors, and these responses reflect the behavior (Lahondère et al., 2020). We suggest that similar results could occur in the neural coding and behavior of these fruit odors. Our experiments manipulating the proportion of select compounds in the different fruit odors show that a subtle change in the emission of a behaviorally relevant compound can modify its attractiveness (Fig. 6). This observation supports the hypothesis that the role of a compound in attraction may depend on its context, such as their proportions within an odor mixture and their percept to the mosquito. Future experiments, using artificial fruit odor mixtures of known constituents and proportions, could further test how altering these proportions influences the olfactory representation and behavioral responses.

An important gap in our current work is the lack of identification of bioactive compounds within the complex fruit odors. Previous work using gas chromatography with electroantennogram detection (GC-EAD) has shown that mosquitoes are responsive to a variety of compounds in the odors of plant nutrient sources. For example, Nyasembe et al. (2018) found that *Ae. aegypti*, *An. gambaie* and *Aedes mcintoshi* mosquitoes detected a similar set of monoterpenes (linalool, linalool oxide, β-myrcene and β-ocimene) in the floral odors. Qualitatively analogous results were found by Lahondère et al. (2020), where *Ae. aegypti, Anopheles stephensi* and *Aedes communis* mosquitoes responded to similar monoterpene and aliphatic aldehyde compounds, such as linalool, lilac aldehyde, β-myrcene, β-ocimene, nonanal and decanal. Future research will be needed to identify which fruit volatiles are detected by the mosquitoes (via GC-EAD, or GC-single sensillum recording) to determine whether the same compounds identified in this study are detected across different mosquito species, and how these odors are encoded in the brain.

Attractant baits incorporating fruit odors are an important control intervention, especially when combined with existing approaches, such as bed nets and insecticides (Njoroge et al., 2023). In limited field trials in Mali, Africa, lures based on Attractive Toxic Sugar Baits (ATSBs^TM^) were able to decrease the number of mosquitoes bearing malaria pathogens (Traore et al., 2020). However, more recent trials using ATSBs^TM^ have shown no efficacy (Steketee, 2025), which raises the question of what might be causing these changes. Mosquito lures such as ATSBs^TM^ often use fruit syrups or fermented fruit juices combined with insecticides to attract and kill feeding mosquitoes (Torto and Tchouassi, 2024; Traore et al., 2020), and variations in the sources of these syrups, such as using different cultivars, could potentially affect their attractiveness and efficacy (Fig. 1B). Syrups and juices also do not incorporate the exocarp, the dominant source of volatiles (Thiruchelvam et al., 2020). In a natural setting, mosquitoes are attracted to whole fruit and damaged fruit on the ground (Joseph, 1970). Formulating exocarp-derived lures or using artificial odors that mimic attractive nutrient sources could increase the attractiveness and longevity of the traps while decreasing sources of variation in the lure's attractiveness.

Beyond the compounds emitted from the exocarp, and beyond the mosquito's sense of smell, plant nutrient sources provide other sensory cues that may attract mosquitoes. For example, the fruits emit high levels of water vapor that attract foraging mosquitoes (Grierson and Wardowski, 1978; Laursen et al., 2023), as well as providing a visually contrasting and spectrally rich display of the nutrient source. Once contacting and tasting the fruit, gustatory stimuli such as sugars, phenolics, terpenes and other antioxidant compounds (Saini et al., 2022) could be potentially detected by the mosquito (Baik and Carlson, 2020). The relative contribution of these other sensory cues in mediating attraction and feeding on plant nutrient sources remains untested. By contrast, a growing number of studies have shown the importance of multiple sensory cues in mediating attraction to blood hosts (McMeniman et al., 2014), including the combination of CO_2_, heat, skin odor, water vapor and/or visual displays (Alonso San Alberto et al., 2022; Chandel et al., 2024; Giraldo et al., 2023; Laursen et al., 2023). Future work will be needed to examine these in more detail for nutrient sources, and determine how the mosquito nervous system detects and processes complex olfactory and multimodal information.

## Supplementary Material

10.1242/jexbio.250305_sup1 Supplementary information
