# Supplementary material for: Olfactory preferences and chemical differences of fruit odors for Aedes aegypti mosquitoes
Source: J Exp Biol. 2025 Sep 26;228(18):jeb250305. doi: 10.1242/jeb.250305 (PMC12517342; doi:10.1242/jeb.250305)
Supplement: Supplementary information [file jexbio-228-250305-s1.pdf]

# Table S1. Fruit information

| Species Name | Cultivar | Variety |
|---|---|---|
| **Mangifera indica* | Tommy Atkins | -- |
| **Mangifera indica* | Keitt | -- |
| **Mangifera indica* | Ataulfo | -- |
| *Prunus persica* | Monroe | -- |
| *Prunus persica* | White Lady | -- |
| **Prunus persica* | Fantasia | nucipersica |
| **Prunus persica* | Snow Queen | nucipersica |
| *Prunus salicinia* | Santa Rosa | -- |
| *Prunus salicinia* | Burgundy | |
| **Psidium guajava* | Pink | -- |
| **Psidium guajava* | White | -- |
| **Musa x paradisiaca* | | -- |
| **Musa acuminata* | Cavendish | -- |
| **Pyrus communis* | Williams | -- |
| **Pyrus pyrifolia* | Korean | -- |
| **Lycopersicon esculentum* | -- | -- |
| **Citrus Limon* | -- | -- |
| **Phoenix dactylifera* | -- | Medjool |
| **Phoenix dactylifera* | -- | Bahri |

**organic fruits*

| Fruit Group | Fruit Information | Mixture Type | Citations |
|---|---|---|---|
| *Mangifera indica* | Kent | Juice | Meza et al 2020 |
| | Mango Goya | Juice | Naranjo et al 2013 |
| | Mango | Mixture | Tenywa et al 2017 |
| *Prunus persica* | Rotting Peach | Mixture | Yu et al 2017 |
| *Prunus persica var nucipersica* | Rotting Nectarine | 75% Juice | Müller et al 2008 |
| | Rotting Nectarine | 85% Juice | Müller et al 2008 |
| | Rotting Nectarine: P | 80% Juice | Müller et al 2010 |
| *Prunus salicinia* | Rotting Plum | Mixture | Müller et al 2010 |
| | Plums | Mixture | Qualls et al 2012 |
| *Psidium guajava* | Guava Juice | Juice | Tenywa et al 2017 |
| | Guava | 35% Juice | Stewart et al 2013 |
| | Guava | 30% Juice | Müller et al 2010 |
| | Guava | 30% Juice | Qualls et al 2012 |
| | Guava | 30 %Juice | Naranjo et al 2013 |
| | Guava | 30% Juice | Müller et al 2010 |
| *Musa x paradisiaca* | Banana | Mixture | Tenywa et al 2017 |
| *Phoenix dactylifera* | Date syrup | Mixture | Junnila et al 2015 |

# Table S2. GCMS Results

| | KRI | *M. indica 'Keitt'* | *M. indica 'TA'* | *M. indica 'Ataulfo'* |
|---|---|---|---|---|
| **Emission rate (ng/h/g):** | | 0.03 | 0.08 | 0.10 |
| **Volatile:** | | | | |
| 3-methylpentan-3-ol | 752 | - | - | 0.42 |
| 2-Hexanone | 789 | 0.04 | 0.02 | 1.11 |
| 3-Hexanone | 792 | 0.40 | 0.04 | 0.63 |
| Cyclopropane, pentyl- | 812 | - | - | - |
| Cyclopentanol, 3-methyl- | 846 | - | - | - |
| **3-Hexen-1-ol** | 847 | - | - | - |
| 3-Methylcyclopentanone | 858 | - | - | - |
| **1-Hexanol** | 876 | - | - | - |
| **Heptanal** | 892 | 0.00 | - | - |
| Nonane | 906 | 0.03 | - | - |
| Ethanol, 2-butoxy- | 910 | - | - | - |
| 2-Penten-1-ol, acetate | 916 | - | - | - |
| 1-Heptanol | 972 | - | - | - |
| **3-Hexen-1-ol, acetate** | 1003 | - | - | 0.20 |
| **Octanal** | 1004 | - | - | - |
| Decane, 4-ethyl- | 1055 | - | - | - |
| **1-Octanol** | 1071 | - | - | 0.13 |
| Decane, 3,6-dimethyl- | 1085 | - | - | - |
| 2-Nonanone | 1094 | - | - | - |
| Undecane | 1100 | 0.34 | - | - |
| **Ethanone, 1-bicyclo[2.2.1]hept-2-en-2-** | 1103 | 0.05 | - | - |
| **Nonanal** | 1104 | - | - | - |
| Dodecane | 1158 | 0.11 | 0.01 | 0.06 |
| 2-Dodecene | 1207 | 0.01 | - | - |
| **Decanal** | 1208 | - | - | - |
| 1-Dodecene | 1209 | - | - | - |
| 2-Isopropyl-5-methyl-1-heptanol | 1299 | - | - | - |
| **Tridecane** | 1300 | 0.24 | - | - |
| 1-Tetradecene | 1392 | - | - | - |
| **Tetradecane** | 1400 | - | - | 0.06 |
| 2-Tetradecene | 1401 | 0.03 | - | - |
| Pentadecane | 1500 | - | - | 0.09 |
| 7-Hexadecene | 1592 | - | - | - |
| **Hexadecane** | 1600 | 0.23 | 0.02 | 0.09 |
| 1-Hexadecene | 1613 | 0.03 | - | - |
| 8-Heptadecene | 1676 | - | - | - |
| **Heptadecane** | 1699 | 0.02 | 0.00 | 0.11 |
| 1-Octadecene | 1786 | - | - | - |
| 5-Octadecene | 1792 | - | - | - |
| **Octadecane** | 1802 | 0.05 | - | - |

Journal of Experimental Biology • Supplementary information

| | | | | |
|---|---|---|---|---|
| 1-Nonadecene | 1862 | 0.01 | - | - |
| Nonadecane | 1900 | 0.01 | - | - |
| Nonadecane, 9-methyl- | 1939 | - | - | - |
| Heneicosane | 2100 | - | - | - |
| Docosane | 2198 | - | 0.07 | 0.07 |
| | | | | |
| **Benzaldehyde** | 967 | - | - | 0.05 |
| **Benzyl Alcohol** | 1044 | - | - | - |
| **Acetophenone** | 1068 | 0.03 | - | - |
| **Benzoic acid, methyl ester** | 1090 | - | - | 0.16 |
| Benzaldehyde, 3-ethyl- | 1166 | - | - | - |
| Acetic acid, phenylmethyl ester | 1168 | - | - | - |
| Benzoic acid, ethyl ester | 1175 | - | - | - |
| Estragole | 1199 | - | - | - |
| **Benzothiozol** | 1218 | - | - | - |
| **Cinnamaldehyde (2-Propenal, 3-pheny** | 1235 | - | - | - |
| **Benzeneacetic acid, ethyl ester (ethyl p** | 1244 | - | - | - |
| Benzenepropanoic acid, methyl ester (M | 1248 | - | - | - |
| 4-Hydroxy-3-methylacetophenone | 1255 | - | - | - |
| 4-ethylacetophenone | 1274 | - | - | - |
| Benzenepropanoic acid, ethyl ester (Eth | 1390 | - | - | - |
| Cinnamyl acetate | 1451 | - | - | - |
| 1,4-Diacetylbenzene | 1460 | - | - | - |
| Hydrocinnamyl isobutyrate | 1500 | - | - | - |
| Benzophenone | 1630 | 0.19 | 0.03 | 0.04 |
| Benzoic acid, 2-ethylhexyl ester | 1712 | - | - | - |
| | | | | |
| Methyl-2-butenoate | 741 | 0.12 | 0.04 | - |
| **Ethyl butyrate** | 785 | 0.10 | 2.34 | - |
| Acetic acid, 2-methylpropyl ester | 788 | - | - | - |
| Butanoic acid, 3-hexenyl ester | 829 | - | - | - |
| Methyl pentanoate | 830 | - | - | - |
| Ethyl but-2-enoate | 841 | 0.38 | 0.53 | - |
| 3-Methyl-3-buten-1-ol, acetate | 861 | - | - | - |
| 2-Methylpropyl propionate | 863 | - | - | - |
| Methyl tiglate | 871 | 0.00 | - | - |
| Formic acid, hexyl ester | 878 | - | - | - |
| 3-Methylbutyl acetate | 879 | - | - | - |
| 2-Methylbutyl acetate | 883 | - | - | - |
| 2-Pentanol, acetate | 902 | - | - | - |
| **Propyl butyrate** | 902 | 0.02 | 0.02 | - |
| Ethyl pentanoate | 903 | - | - | - |
| Methyl 3-hexenoate | 916 | - | - | 0.01 |
| Acetic acid, pentyl ester | 921 | - | - | - |

Journal of Experimental Biology • Supplementary information

| | | | | |
|---|---|---|---|---|
| **Methyl hexanoate** | 929 | 0.14 | 0.07 | 0.15 |
| **Ethyl tiglate** | 962 | 0.06 | 0.11 | - |
| Ethyl 4-hexenoate | 986 | - | - | - |
| **Ethyl hexanoate** | 1001 | 2.23 | 6.84 | 0.76 |
| Ethyl 3-hexenoate | 1002 | 0.02 | 0.01 | - |
| Ethyl-5-hexenoate | 1002 | - | - | - |
| Ethyl 2-hexenoate | 1006 | 0.09 | 0.12 | - |
| Acetic acid, hexyl ester | 1023 | - | - | 0.11 |
| Propyl hexanoate | 1084 | 0.07 | - | - |
| Ethyl heptanoate | 1093 | 0.02 | 0.08 | 0.17 |
| 4-Heptenoic acid, ethyl ester | 1096 | - | - | - |
| **Methyl octanoate** | 1125 | 1.51 | 0.21 | 1.72 |
| Acetic acid, 2-ethylhexyl ester | 1146 | - | - | - |
| Methyl-2-octenoate | 1170 | 0.14 | - | - |
| 4-Octenoic acid, ethyl ester | 1183 | 0.43 | 0.26 | 0.66 |
| Hexyl butyrate (Butanoic acid, hexyl est | 1190 | - | - | - |
| **Ethyl octanoate** | 1194 | 7.31 | 16.10 | 4.96 |
| Methyl nonanoate | 1204 | - | - | - |
| Ethyl 2-octenoate | 1226 | 0.14 | 0.08 | - |
| 2-Ethylhexyl butyrate | 1304 | - | - | - |
| 4-Decenoic acid, methyl ester | 1306 | 0.60 | - | 0.21 |
| Methyl decanoate (Decanoic acid, methy | 1322 | 0.20 | - | - |
| Ethyl trans-4-decenoate | 1361 | 0.51 | 0.94 | 0.25 |
| Methyl-2,4-decadienoate | 1389 | - | - | - |
| Ethyl decanoate | 1396 | 0.16 | 0.24 | - |
| 3-Hexenyl hexanoate | 1397 | - | - | - |
| Methyl dodeconoate | 1524 | 0.52 | - | - |
| Octyl hexanoate | 1579 | - | - | - |
| Ethyl dodeconoate | 1591 | 0.42 | 0.07 | - |
| Ethyl tetradecanoate | 1728 | - | 0.01 | - |
| Methyl hexadecanoate | 1923 | 0.05 | - | - |
| Ethyl 11-hexadecenoate | 1970 | - | - | - |
| Ethyl hexadecanoate | 1997 | - | - | - |
| Ethyl Oleate | 2156 | - | - | - |
| | | | | |
| Furan, 2-pentyl | 992 | - | - | - |
| 3(2H)-Furanone, 4-methoxy-2,5-dimeth | 1046 | - | - | - |
| | | | | |
| **alpha.-Pinene** | 938 | 1.46 | - | 39.54 |
| **Camphene** | 953 | 0.06 | 1.04 | 0.39 |
| α-Fenchene | 953 | 0.07 | - | 0.03 |
| **beta-Pinene** | 972 | - | 4.94 | 2.27 |
| **beta-Thujene** | 973 | 1.94 | 1.66 | 0.08 |
| **Sabinene** | 977 | - | - | 0.03 |

Journal of Experimental Biology • Supplementary information

| | | | | |
|---|---|---|---|---|
| **beta-Myrcene** | 991 | 0.17 | 0.44 | 0.17 |
| **3-Carene** | 1008 | 61.32 | 57.61 | 43.20 |
| **α-Terpinene** | 1020 | 0.31 | - | 0.19 |
| **Eucalyptol** | 1035 | - | - | - |
| **Limonene** | 1035 | 2.46 | 3.27 | 1.26 |
| **beta-Phellandrene** | 1039 | 0.44 | 0.71 | 0.25 |
| γ-Terpinene | 1060 | 0.05 | 0.39 | 0.08 |
| 7-Octen-2-ol, 2,6-dimethyl- | 1072 | - | - | - |
| **Linalool** | 1099 | - | - | - |
| **Limonene oxide** | 1116 | 0.05 | - | - |
| **Eucarvone** | 1238 | 0.08 | 0.00 | - |
| Longipinene | 1342 | 0.01 | - | - |
| alpha-Cubebene | 1363 | - | - | - |
| beta-Panasinsene | 1376 | 0.03 | - | - |
| Copaene | 1384 | 0.22 | 0.19 | - |
| **Bergamotene** | 1433 | 0.02 | - | - |
| **Caryophyllene** | 1435 | 4.28 | 0.30 | 0.22 |
| 1,4,7,-Cycloundecatriene, 1,5,9,9-tetram | 1466 | 2.24 | - | 0.08 |
| **β-Ionone** | 1485 | - | - | - |
| **Germacrene D** | 1491 | 5.11 | 1.19 | - |
| 1H-Benzocycloheptene, 2,4a,5,6,7,8-he: | 1496 | 0.53 | - | - |
| **alpha-Farnesene** | 1504 | 1.73 | - | - |
| alpha-Panasinsen | 1529 | 0.39 | - | 0.00 |
| **Caryophyllene oxide** | 1585 | - | - | - |
| | | | | |
| **Disulfide, dimethyl** | 692 | - | - | - |
| **Dimethyl trisulfide** | 954 | - | - | - |

Journal of Experimental Biology • Supplementary information

| *P. guajava 'Pink'* | *P. guajava 'White'* | *P. persica 'Monroe'* | *P. persica 'White Lad* | *P. salicinia 'Santa Ro* | *P. salicinia 'Burgund* |
|---|---|---|---|---|---|
| 0.50 | 0.04 | 0.08 | 0.03 | 0.05 | 0.07 |
| | | | | | |
| 0.00 | - | - | - | - | - |
| 0.01 | 0.08 | 1.08 | - | - | 0.03 |
| 0.03 | 0.36 | 0.77 | - | - | 0.65 |
| 0.66 | 1.48 | 0.39 | - | - | 0.20 |
| - | - | - | - | - | - |
| 0.24 | 1.41 | - | - | - | - |
| - | - | - | - | - | - |
| 0.07 | - | - | - | - | - |
| - | - | - | - | - | - |
| - | - | - | - | - | - |
| - | - | - | - | - | - |
| 0.02 | 0.03 | 0.01 | - | - | - |
| - | - | - | - | - | - |
| 11.27 | 33.00 | 2.66 | - | - | - |
| - | - | - | - | - | - |
| 0.26 | 1.01 | - | - | - | 0.16 |
| 0.04 | - | 0.92 | 2.56 | 4.09 | - |
| | | | | | |
| 0.01 | - | 0.01 | - | - | 0.08 |
| 0.02 | 0.43 | - | - | - | - |
| - | - | - | 0.29 | - | - |
| - | - | - | - | - | - |
| 0.02 | - | - | - | - | - |
| - | 0.14 | 2.06 | 1.79 | 1.49 | 0.93 |
| - | - | - | - | - | - |
| - | - | - | - | 0.10 | 0.07 |
| - | - | - | - | 0.06 | - |
| - | - | - | - | - | - |
| - | - | - | 1.10 | 0.36 | - |
| - | - | - | - | 0.18 | - |
| - | 0.14 | 1.34 | 12.83 | 7.35 | 7.85 |
| 0.06 | - | - | - | 5.57 | 0.45 |
| 0.08 | 0.22 | 1.65 | 5.51 | - | 0.40 |
| 0.03 | - | 0.03 | - | 0.16 | - |
| 0.01 | - | 5.85 | 14.26 | 6.81 | 7.75 |
| - | - | - | - | 0.50 | 1.21 |
| 0.04 | - | - | - | - | - |
| 1.33 | 0.37 | 7.03 | 7.22 | 23.38 | 8.85 |
| 0.00 | - | - | - | - | - |
| - | - | 2.56 | - | 1.78 | - |
| 0.01 | 0.03 | 7.95 | 5.44 | 4.81 | 9.06 |

Journal of Experimental Biology • Supplementary information

| | | | | | |
|---|---|---|---|---|---|
| - | - | - | - | 0.41 | 2.55 |
| 0.04 | 0.16 | 4.87 | 1.75 | 8.79 | 3.46 |
| - | - | - | - | 0.52 | 2.89 |
| 0.25 | 0.01 | 0.78 | 2.85 | 9.67 | 0.91 |
| 0.03 | 0.11 | 0.13 | 4.16 | 0.62 | 9.31 |
| | | | | | |
| 0.02 | 0.68 | 0.01 | - | - | 0.04 |
| 0.03 | 0.08 | - | - | 2.77 | 1.15 |
| 0.01 | 0.48 | 1.34 | 0.29 | - | 0.04 |
| 5.94 | - | - | - | - | - |
| 0.09 | - | - | - | - | - |
| 0.38 | - | - | - | - | - |
| 6.29 | 0.03 | - | - | - | - |
| 0.03 | - | - | - | - | - |
| - | - | - | - | - | - |
| 0.10 | 0.05 | - | - | - | - |
| 0.04 | - | - | - | - | - |
| 0.12 | - | - | - | - | - |
| - | - | - | - | - | - |
| - | - | - | - | - | - |
| 0.49 | - | - | - | - | - |
| 4.94 | - | - | - | - | - |
| - | - | - | - | - | - |
| 3.27 | - | - | - | - | - |
| - | 0.01 | 2.64 | - | 1.20 | 1.07 |
| - | - | 2.91 | - | 0.17 | - |
| | | | | | |
| - | - | - | - | - | - |
| 30.19 | 12.88 | 0.04 | - | 0.80 | - |
| 0.16 | 0.15 | 0.02 | - | - | - |
| 0.88 | - | - | - | - | - |
| 0.01 | - | - | - | - | - |
| 0.10 | - | - | - | - | - |
| 0.00 | - | - | - | - | - |
| 0.00 | - | - | - | - | - |
| - | - | - | - | - | - |
| 0.01 | - | - | - | - | - |
| 0.35 | 0.06 | 0.02 | 1.10 | - | - |
| 0.12 | - | - | - | - | - |
| 0.04 | - | - | - | - | - |
| 0.02 | - | - | - | - | - |
| 0.57 | 0.05 | - | - | - | - |
| 0.01 | - | 0.02 | - | - | - |
| 0.02 | - | 0.04 | 0.75 | - | - |

Journal of Experimental Biology • Supplementary information

| | | | | | |
|---|---|---|---|---|---|
| 2.29 | 0.10 | 0.17 | - | - | - |
| - | - | - | - | - | - |
| - | - | - | - | 0.18 | - |
| 11.87 | 1.59 | 0.53 | 1.74 | 1.71 | - |
| - | - | - | - | - | - |
| 0.02 | - | - | - | - | - |
| 0.11 | - | - | - | - | - |
| 2.16 | 3.20 | 0.76 | 0.62 | - | - |
| - | - | - | - | - | - |
| - | - | 1.57 | 1.72 | - | - |
| 0.01 | - | - | - | - | - |
| 9.21 | - | 21.31 | 1.74 | - | - |
| 0.08 | 1.12 | 1.16 | - | - | - |
| - | - | 0.06 | - | - | - |
| 0.49 | - | 4.22 | - | - | - |
| 0.07 | - | - | - | 0.86 | - |
| 2.77 | - | 12.48 | 14.84 | 0.94 | - |
| 0.01 | - | 0.01 | - | - | - |
| - | - | - | - | - | - |
| 0.01 | - | - | - | - | - |
| - | - | 3.04 | - | - | - |
| - | - | 0.05 | - | - | - |
| - | - | 5.19 | - | 0.36 | - |
| 0.01 | - | - | - | - | - |
| 0.09 | - | - | - | 0.93 | - |
| 0.05 | - | - | - | 0.35 | - |
| - | - | 0.21 | - | 1.44 | - |
| 0.00 | - | - | - | - | - |
| - | - | - | - | - | - |
| 0.06 | - | - | - | 3.40 | - |
| 0.02 | 12.82 | 1.73 | 13.09 | 1.58 | 37.04 |
| 0.01 | - | - | - | - | - |
| 0.14 | - | 0.01 | - | - | - |
| 0.00 | - | - | - | - | - |
| | | | | | |
| - | - | - | - | - | - |
| 0.02 | - | - | - | - | - |
| | | | | | |
| - | - | - | 1.92 | 1.60 | 0.05 |
| 0.01 | 0.04 | - | 0.44 | 0.81 | 0.10 |
| - | - | - | - | - | - |
| 0.01 | - | - | - | - | - |
| - | - | - | - | - | - |
| 0.01 | - | - | - | - | - |

Journal of Experimental Biology • Supplementary information

| | | | | | |
|---|---|---|---|---|---|
| 0.01 | 0.04 | - | - | 0.41 | 1.53 |
| - | - | - | - | - | - |
| - | - | - | - | - | - |
| - | - | - | - | - | - |
| 0.09 | 0.91 | 0.02 | 1.98 | 2.25 | 2.17 |
| 0.14 | - | - | - | - | - |
| - | - | - | - | - | - |
| - | - | - | - | - | - |
| - | - | - | - | - | - |
| - | - | - | - | - | - |
| - | - | - | - | - | - |
| - | - | - | - | - | - |
| 0.02 | 2.33 | - | - | - | - |
| - | - | - | - | - | - |
| - | 0.77 | - | - | - | - |
| - | - | - | - | - | - |
| 0.12 | 20.47 | - | - | - | - |
| - | 0.82 | - | - | - | - |
| 1.29 | - | - | - | - | - |
| - | - | - | - | - | - |
| - | - | - | - | - | - |
| - | - | 0.35 | - | 1.32 | - |
| - | - | - | - | 0.25 | - |
| 0.03 | 2.34 | - | - | - | - |
| | | | | | |
| 0.03 | - | - | - | - | - |
| 0.02 | - | - | - | - | - |

Journal of Experimental Biology • Supplementary information

| ***P. persica nucipersica*** | ***P. persica nucipersica*** | ***M. paradisiaca*** | ***M. acuminata 'Caven*** | ***P. pyrifolia*** | ***P. communis*** |
|---|---|---|---|---|---|
| 0.01 | 0.01 | 0.06 | 0.11 | 0.01 | 0.37 |
| | | | | | |
| - | - | - | - | - | - |
| - | 0.54 | - | - | - | - |
| - | - | - | - | - | 0.00 |
| - | - | - | - | - | 0.22 |
| - | - | - | - | - | - |
| 1.02 | 1.87 | - | - | - | - |
| - | - | - | - | - | - |
| 4.67 | 3.58 | - | - | - | 0.89 |
| - | - | 0.01 | - | - | - |
| - | - | 0.02 | - | - | - |
| 0.11 | - | - | - | - | - |
| - | - | - | - | - | - |
| 0.08 | - | - | - | - | - |
| 2.11 | - | 0.25 | - | - | - |
| - | 0.98 | - | - | - | - |
| - | - | 0.17 | - | - | - |
| 0.06 | - | 12.87 | - | - | - |
| | | | | | |
| - | - | 0.04 | - | - | - |
| 0.04 | - | 0.02 | - | - | - |
| - | - | - | - | - | - |
| 5.92 | 54.00 | - | - | - | - |
| 1.08 | 8.86 | - | - | - | - |
| 0.78 | 0.84 | 2.54 | - | 8.89 | - |
| - | - | - | - | - | - |
| - | - | - | - | - | - |
| - | - | - | - | - | - |
| - | - | - | - | - | - |
| - | - | - | - | - | 0.02 |
| 0.10 | - | - | - | - | - |
| 6.20 | 5.19 | 3.39 | 0.21 | 13.90 | - |
| - | - | - | - | - | - |
| 2.20 | 2.79 | - | - | - | - |
| 0.24 | - | - | - | - | - |
| 4.36 | 0.54 | 23.56 | - | 10.19 | 0.10 |
| - | - | - | - | - | - |
| - | - | - | - | - | 0.00 |
| 1.85 | 2.91 | - | - | - | - |
| - | - | - | - | - | - |
| - | - | - | - | - | - |
| 0.91 | 1.20 | 1.67 | 0.05 | - | 0.01 |

Journal of Experimental Biology • Supplementary information

| | | | | | |
|---|---|---|---|---|---|
| - | - | - | - | - | - |
| 0.08 | 0.45 | 0.03 | - | - | - |
| - | - | - | - | - | - |
| 1.03 | - | - | - | - | - |
| 0.07 | 0.51 | 0.02 | - | - | - |
| | | | | | |
| 4.23 | 7.42 | 0.13 | - | - | 0.01 |
| 0.06 | 0.58 | - | - | - | - |
| 0.14 | - | 0.05 | - | - | 0.01 |
| - | - | - | - | - | - |
| - | - | - | - | - | - |
| - | - | - | - | - | - |
| - | - | 0.09 | - | - | - |
| - | - | - | - | - | 2.90 |
| - | 1.34 | - | - | - | - |
| - | - | 4.79 | - | - | - |
| - | - | - | - | - | - |
| - | - | - | - | - | - |
| 0.27 | - | - | - | - | - |
| - | - | - | - | - | - |
| - | - | - | - | - | - |
| - | - | - | - | - | - |
| - | - | - | - | - | - |
| - | - | - | - | - | - |
| - | 1.82 | 0.84 | 0.11 | - | - |
| - | - | - | - | - | - |
| | | | | | |
| - | - | - | - | - | - |
| - | - | - | 0.09 | - | 0.07 |
| - | - | 0.49 | 6.24 | - | 0.02 |
| - | - | - | 0.51 | - | - |
| - | - | - | - | - | - |
| - | - | - | - | - | - |
| - | - | - | - | - | - |
| - | - | 6.45 | 25.65 | - | 0.01 |
| - | - | - | - | - | - |
| - | - | - | - | - | - |
| - | - | 6.78 | 40.54 | - | 0.02 |
| 0.05 | - | - | - | - | 0.10 |
| 0.09 | - | 6.91 | 20.32 | - | - |
| - | - | - | 0.14 | - | 0.05 |
| 0.34 | - | - | - | - | - |
| - | - | - | - | - | - |
| - | - | - | - | - | - |

Journal of Experimental Biology • Supplementary information

| | | | | | |
|---|---|---|---|---|---|
| - | - | - | - | - | 0.03 |
| - | - | - | - | - | - |
| - | - | - | - | - | - |
| 1.26 | - | - | - | - | - |
| - | - | - | - | - | - |
| - | - | - | - | - | - |
| - | - | - | - | - | - |
| 4.34 | - | - | - | - | 57.64 |
| - | - | - | - | - | - |
| 0.23 | - | - | - | - | 0.02 |
| - | - | - | - | - | - |
| 0.78 | 1.41 | - | - | - | - |
| - | - | - | - | - | - |
| - | - | - | - | - | - |
| - | - | - | - | - | - |
| - | - | 0.20 | 6.13 | - | 5.14 |
| 2.41 | - | - | - | - | 0.39 |
| - | - | - | - | - | - |
| - | - | - | - | - | 0.26 |
| - | - | - | - | - | - |
| - | - | - | - | - | 0.04 |
| - | - | - | - | - | 0.04 |
| - | - | - | - | - | - |
| - | - | - | - | - | - |
| - | - | - | - | - | - |
| - | - | - | - | - | - |
| - | - | - | - | - | 0.04 |
| - | - | - | - | - | - |
| - | - | - | - | - | - |
| - | - | - | - | - | 0.02 |
| - | - | 0.02 | - | - | 0.39 |
| - | - | - | - | - | - |
| - | - | - | - | - | 0.11 |
| - | - | - | - | - | 0.22 |
| | | | | | |
| 1.33 | 1.30 | - | - | - | - |
| 5.10 | 0.45 | - | - | - | - |
| | | | | | |
| 1.59 | 0.10 | 0.26 | - | - | - |
| - | - | 1.32 | - | - | - |
| 1.18 | - | - | - | - | - |
| - | - | - | - | - | - |
| 0.05 | - | - | - | - | - |
| - | - | - | - | - | - |

Journal of Experimental Biology • Supplementary information

| | | | | | |
|---|---|---|---|---|---|
| - | - | 3.09 | - | - | 0.01 |
| - | - | - | - | - | - |
| - | - | - | - | - | - |
| 25.01 | - | - | - | - | - |
| 0.40 | 0.47 | 11.27 | - | - | 0.11 |
| 1.86 | 0.83 | 0.04 | - | - | - |
| - | - | - | - | - | - |
| 0.10 | - | - | - | - | - |
| 1.05 | - | - | - | - | - |
| - | - | - | - | - | - |
| - | - | - | - | - | - |
| - | - | - | - | - | - |
| - | - | - | - | - | 0.01 |
| - | - | - | - | - | - |
| - | - | 0.10 | - | - | 0.28 |
| - | - | - | - | - | - |
| 1.05 | - | - | - | - | 0.09 |
| - | - | 0.59 | - | - | - |
| - | - | 9.17 | - | - | - |
| - | - | 1.41 | - | - | - |
| - | - | - | - | - | - |
| 14.18 | - | - | - | 67.02 | 30.72 |
| - | - | - | - | - | - |
| - | - | 1.43 | - | - | - |
| | | | | | |
| - | - | - | - | - | - |
| - | - | - | - | - | - |

Journal of Experimental Biology • Supplementary information

| ***P. dactylifera 'Medjool*** | ***P. dactylifera 'Bahri'*** |
|---|---|
| 0.01 | 0.01 |
| | |
| 0.18 | - |
| 1.32 | - |
| 1.64 | 0.20 |
| 7.95 | - |
| 1.61 | 2.73 |
| - | - |
| 0.64 | - |
| 4.44 | 0.19 |
| - | - |
| - | - |
| - | 3.92 |
| - | - |
| - | 3.38 |
| - | - |
| 0.15 | 0.68 |
| - | - |
| - | - |
| | |
| - | - |
| - | - |
| - | - |
| - | - |
| 9.27 | 8.76 |
| 0.16 | - |
| - | - |
| - | 0.17 |
| - | - |
| 1.62 | 12.21 |
| - | - |
| - | - |
| 0.31 | - |
| - | - |
| - | 1.63 |
| - | - |
| 1.69 | 0.56 |
| - | - |
| - | - |
| - | - |
| - | - |
| - | - |
| - | - |

Journal of Experimental Biology • Supplementary information

| | |
|---|---|
| - | - |
| - | - |
| - | - |
| - | - |
| - | - |
| | |
| 1.48 | 2.98 |
| - | - |
| 0.59 | 7.35 |
| - | - |
| 6.05 | 13.48 |
| - | - |
| - | - |
| - | - |
| 0.68 | 3.31 |
| - | - |
| - | - |
| - | - |
| 11.05 | - |
| 43.28 | 37.79 |
| - | - |
| - | - |
| 0.57 | - |
| - | - |
| - | - |
| - | - |
| | |
| - | - |
| - | - |
| - | - |
| - | - |
| - | - |
| - | - |
| - | - |
| - | - |
| - | - |
| - | - |
| - | - |
| - | - |
| - | - |
| - | - |
| - | - |
| - | - |
| - | - |

| | |
|---|---|
| - | - |
| - | - |
| - | - |
| - | - |
| - | - |
| - | - |
| - | - |
| - | - |
| - | - |
| - | - |
| - | - |
| - | - |
| - | - |
| - | - |
| - | - |
| - | - |
| - | - |
| - | - |
| - | - |
| - | - |
| - | - |
| - | - |
| - | - |
| - | - |
| - | - |
| - | - |
| - | - |
| - | - |
| - | - |
| - | - |
| - | - |
| - | - |
| - | - |
| - | - |
| | |
| - | - |
| - | - |
| | |
| 5.14 | 0.67 |
| - | - |
| - | - |
| - | - |
| - | - |
| - | - |

Journal of Experimental Biology • Supplementary information

| | |
|---|---|
| - | - |
| - | - |
| - | - |
| 0.17 | - |
| - | - |
| - | - |
| - | - |
| - | - |
| - | - |
| - | - |
| - | - |
| - | - |
| - | - |
| - | - |
| - | - |
| - | - |
| - | - |
| - | - |
| - | - |
| - | - |
| - | - |
| - | - |
| - | - |
| - | - |
| | |
| - | - |
| - | - |

Journal of Experimental Biology • Supplementary information
